# Long-term survival of an adult patient with undifferentiated embryonal sarcoma of the liver with multidisciplinary treatment: a case report and literature review

**DOI:** 10.1186/s40792-022-01436-3

**Published:** 2022-05-05

**Authors:** Yukiko Kumata Endo, Atsushi Fujio, Keigo Murakami, Kengo Sasaki, Koji Miyazawa, Toshiaki Kashiwadate, Kazuaki Tokodai, Shigehito Miyagi, Fumiyoshi Fujishima, Michiaki Unno, Takashi Kamei

**Affiliations:** 1 Department of Surgery, Tohoku University Graduate School of Medicine, Tohoku University, 1-1 Seiryo-machi, Aoba-ku, Sendai, Miyagi 980-8574 Japan; 2 Division of Pathology, Faculty of Medicine, Tohoku Medical and Pharmaceutical University, 1-15-1 Fukumuro, Miyagino-ku, Sendai, Miyagi 983-8536 Japan; 3 Department of Pathology, Tohoku University, 1-1 Seiryo-machi, Aoba-ku, Sendai, Miyagi 980-8574 Japan

**Keywords:** Undifferentiated embryonal sarcoma of the liver, Long-term survival, Multidisciplinary treatment, Discovered on gastrointestinal stromal tumor-1

## Abstract

**Background:**

Undifferentiated embryonal sarcoma of the liver (UESL) primarily occurs in children; it is rarely seen in adults and appears to have a poor prognosis. However, in recent years, some cases indicated that long-term survival was possible due to a combination of multiple surgeries, chemotherapy, and liver transplantation.

**Case presentation:**

A 33-year-old female patient presented with a complaint of epigastric pain, for which she underwent a medical examination. Computed tomography (CT) and magnetic resonance imaging showed a cystic tumor in the right hepatic lobe, approximately 10 cm in size. During observation, the abdominal pain worsened, and a contrast-enhanced CT revealed that the tumor’s peripheral solid components increased in size and volume, suggesting a malignant tumor threatening hepatic rupture. Subsequently, transcatheter arterial embolization of the anterior and posterior segmental branches of the hepatic artery was performed, followed by right trisectionectomy. Histopathological and immunohistochemical examinations of the lesion revealed UESL. Two months after the surgery, we initiated sarcoma-directed chemotherapy with doxorubicin because of multiple metastases to the liver. After initiating the chemotherapy, she received another regimen using gemcitabine/docetaxel, eribulin, trabectedin, ifosfamide/mesna, pazopanib, and cisplatin. During the chemotherapy, she underwent palliative surgery twice due to the progressive disease. She lived for 49 months after the initial operation.

**Conclusions:**

Improved long-term survival was achieved in an adult patient with UESL after multidisciplinary therapy, involving a combination of three surgical procedures and several chemotherapies.

## Background

Undifferentiated embryonal sarcoma of the liver (UESL) is a malignant mesenchymal tumor that occurs predominantly in juveniles aged 6–10 years [1]. Since UESL is rare in adults [1] and often asymptomatic, the diagnosis at an early stage is challenging, and the prognosis is very poor. Although the standard treatment for UESL had not yet been established, previous literature suggested that a multidisciplinary approach involving surgery, chemotherapy, and radiation therapy could improve the prognosis. Herein, we report improved long-term survival in an adult patient with UESL and reviewed related literature.

## Case presentation

**Fig. 1:**
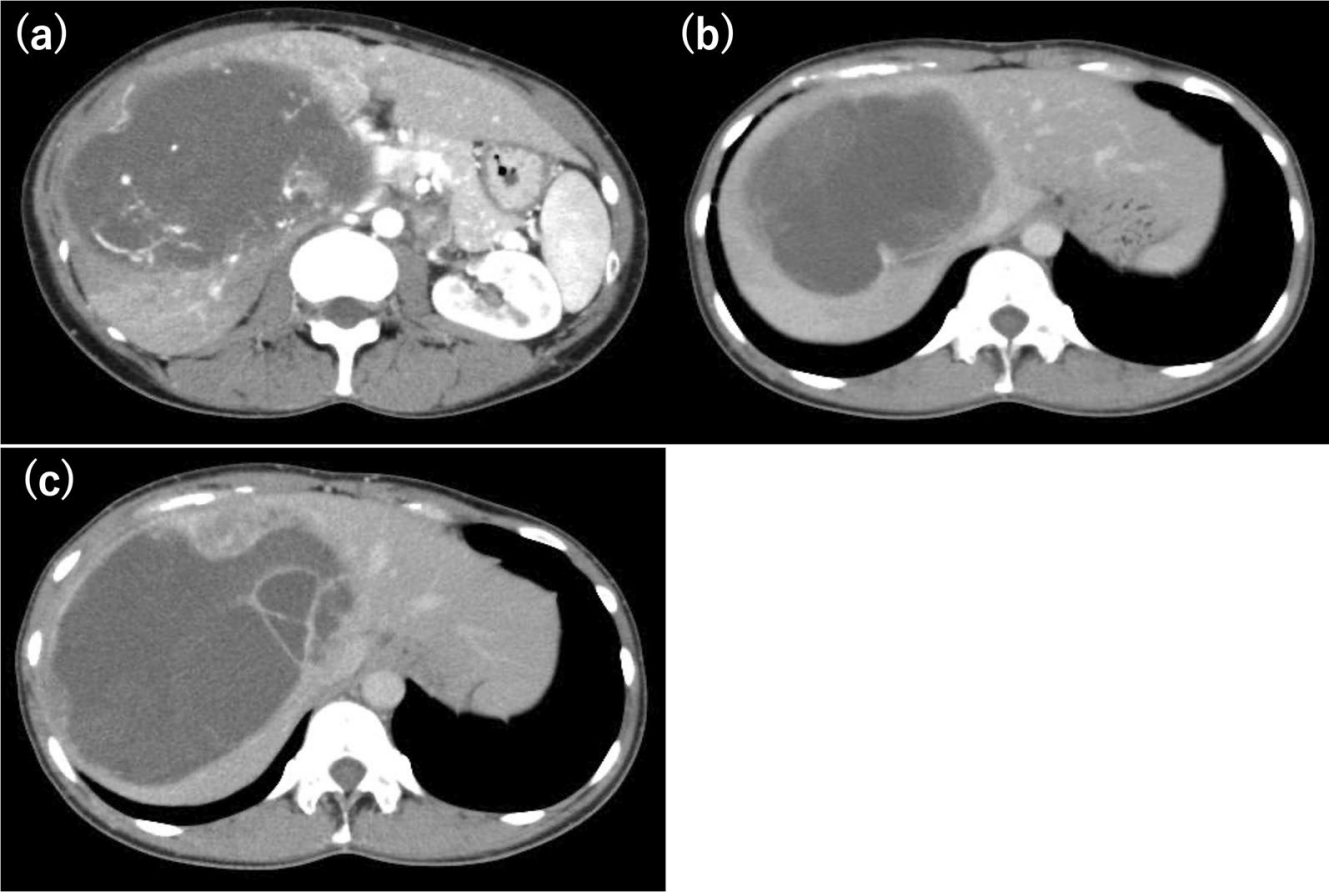
**a** and **b** Computed tomography showing a well-defined cyst. **c** At the time of exacerbation of abdominal pain, the tumor grows and its solid component is increasing

**Fig. 2:**
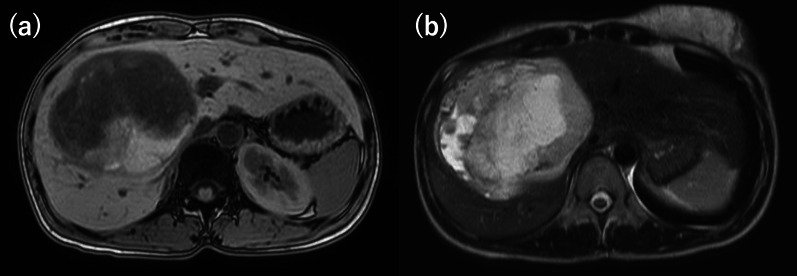
**a** Magnetic resonance imaging reveals a hypointense multicystic tumor on T1-weighted and b hyperintense on T2-weighted images

**Fig. 3:**
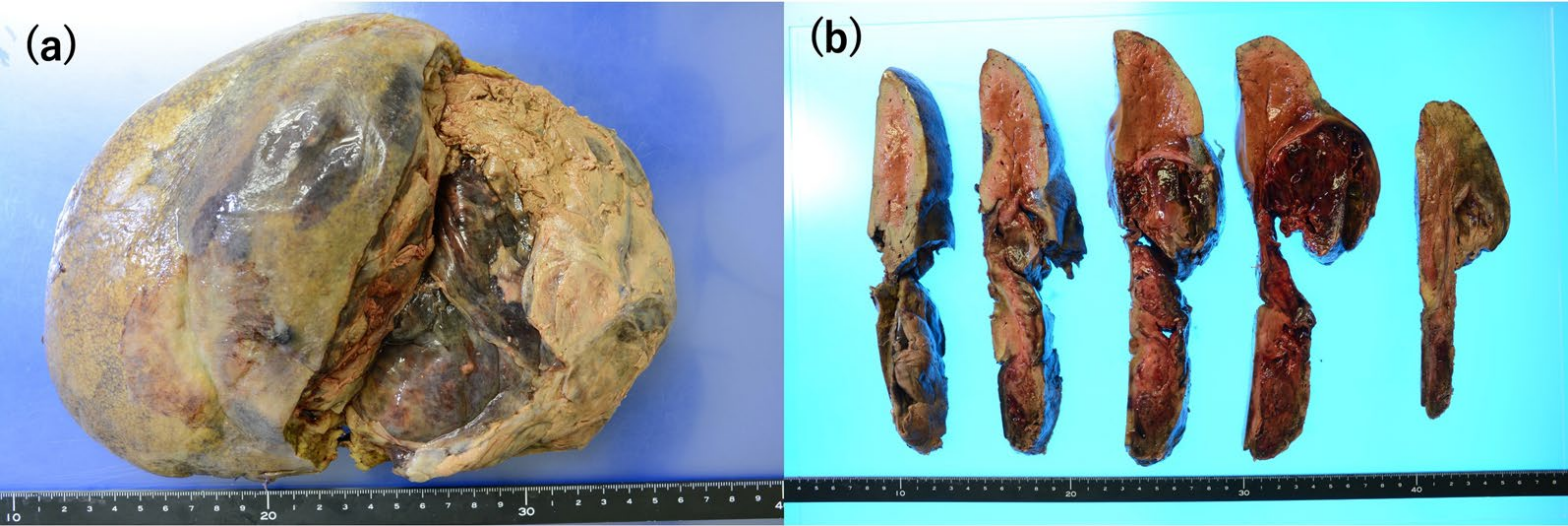
**a** and **b** A resected specimen reveals a well-encapsulated cyst with mural nodules

**Fig. 4:**
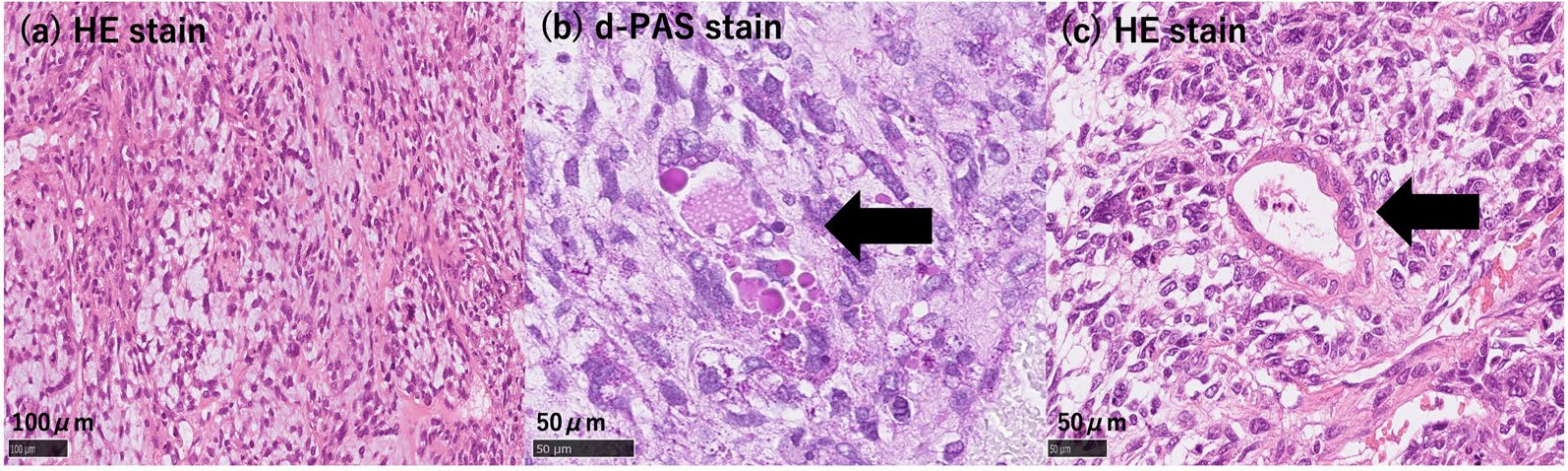
**a** The tumor is composed of spindle-shaped cells (× 200, HE stain). **b** Some tumor cells contain eosinophilic globules, which are d-PAS-positive (× 400, PAS stain, black arrow). **c** Sarcomatous cells surround bile duct-like structures (× 400, HE stain, black arrow). d-PAS, d-periodic acid Schiff; HE, hematoxylin and eosin

**Fig. 5:**
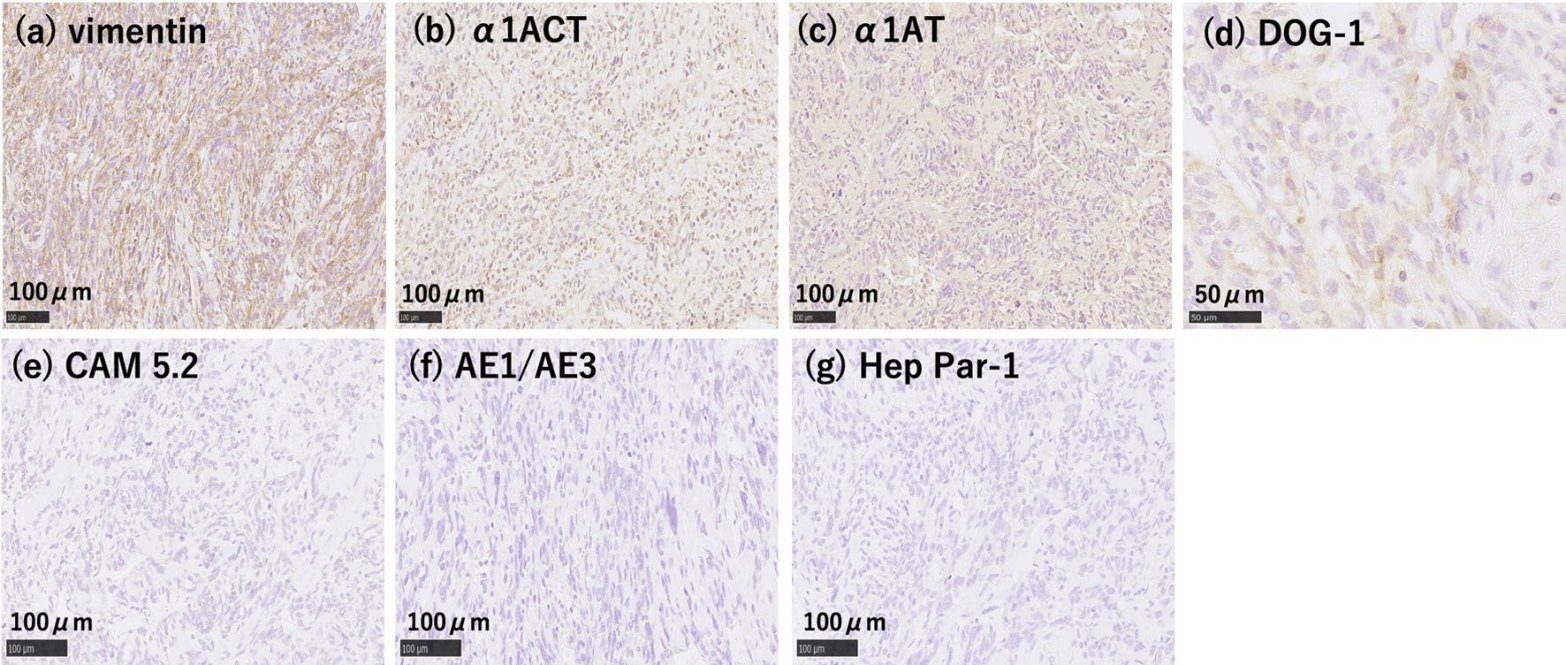
**a** Immunohistochemical analysis reveals that the tumor is stained with vimentin; **b** α1ACT; **c** α1AT (× 200); and **d** focally stained with DOG-1 (× 400) and negative for CAM 5.2 (**e**), AE1/AE3 (**f**), and Hep Par-1 (**g**) (× 200). *α1ACT* alpha 1-antichymotrypsin, *α1AT* alpha 1-antitrypsin, *DOG-1* discovered on gastrointestinal stromal tumor-1, *Hep Par-1* hepatocyte paraffin-1

**Fig. 6:**
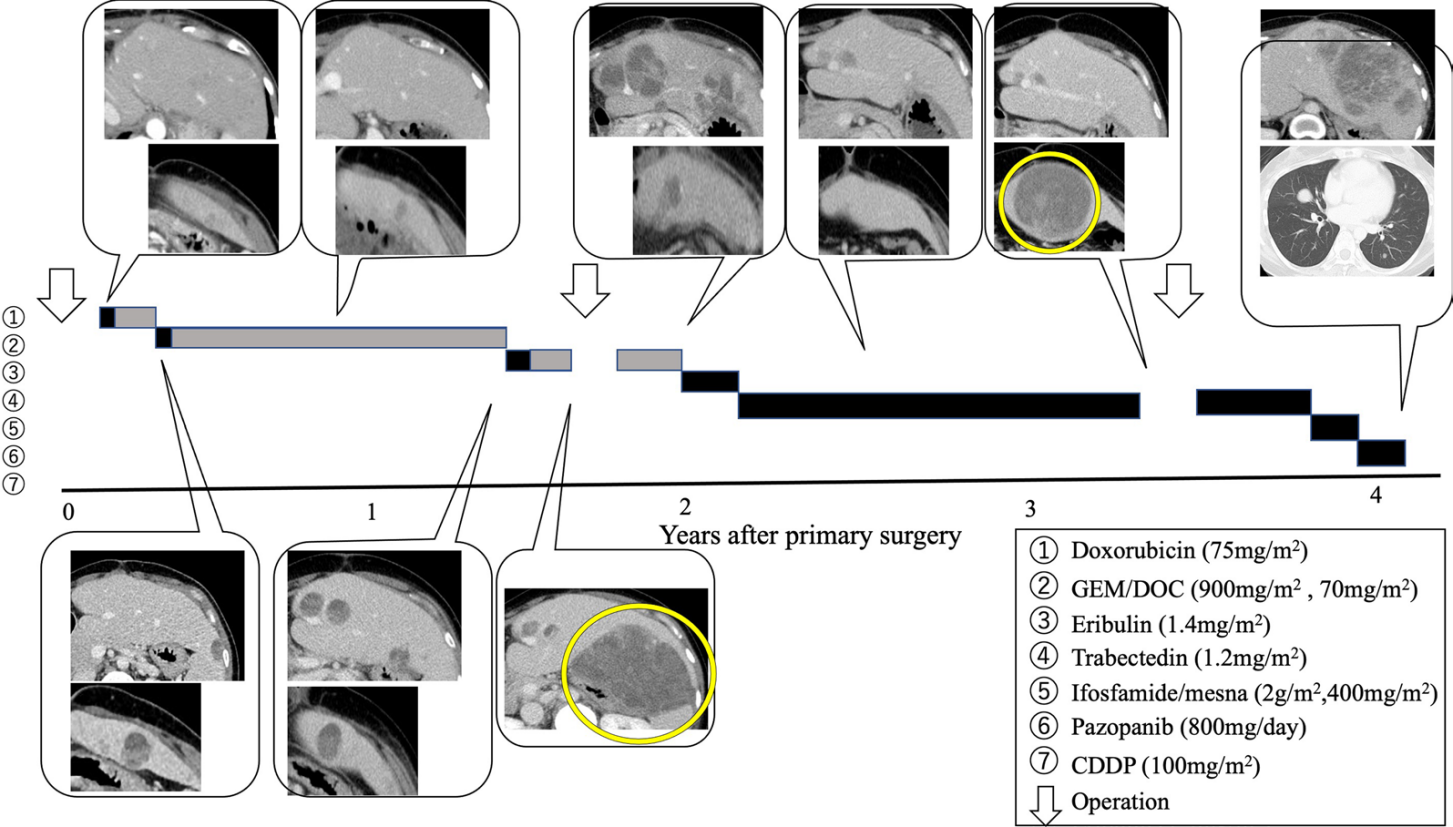
This figure shows the progress in chemotherapy (black bars). Gray bars indicate drug dose reductions. In the second and third operations, palliative partial hepatectomy of one rapidly growing tumor (yellow circles) is performed. The patient is administered doxorubicin (75 mg/m^2^/dose every 3 weeks for 3 cycles); gemcitabine (900 mg/m^2^/dose, days 1 and 8) and docetaxel (70 mg/m^2^/dose every 3 weeks for 16 cycles, day 8); eribulin (1.4 mg/m^2^/dose every 3 weeks for 6 cycles, days 1 and 8); trabectedin (1.2 mg/m^2^/dose every 3 weeks for 3 cycles); ifosfamide (2 g/m^2^/dose, days 1–5) and mesna (400 mg/m^2^/dose given 4 weeks for 15 cycles, days 1–5); pazopanib (800 mg/day dose given 4 weeks for 2 cycles); and CDDP (100 mg/m^2^/dose given 3 weeks for 3 cycles). *GEM* gemcitabine, *DOC* docetaxel, *CDDP* cisplatin

A 33-year-old woman with epigastric pain was identified to have a 10-cm cystic mass in the right lobe of her liver on imaging. Upper and lower gastrointestinal endoscopy and whole-body computed tomography (CT) scan did not identify any primary tumor, except that in the liver. Laboratory tests showed a normal serum bilirubin level, slightly decreased hemoglobin level (10.4 g/dl), and insignificantly increased values of hepatobiliary enzymes, including alkaline phosphatase (359 U/L), γ-glutamyl transpeptidase γ-(GTP) (58 U/L), lactate dehydrogenase (494 U/L), aspartate aminotransferase (35 U/L), and alanine transferase (31 U/L). Studies for hepatitis B and C viral markers were negative. Tests for tumor markers, including carcinoembryonic antigen, α-fetoprotein, cancer antigen 19–9, and protein induced by vitamin K absence-II, were within normal range. Abdominal pain got worse several months after follow-up, and the CT scan revealed that the tumor had enlarged, nearing imminent rupture (Figs. 1, 2). Immediately after the urgent transcatheter arterial embolization (TAE), she was transferred to our hospital. Right hepatic trisectionectomy was performed to treat the tumor. The resected specimen revealed a cystic tumor, weighing 1985 g and 17 × 15 × 15 cm in size, composed of dark reddish hemorrhage and grayish-white solid lesion. The tumor border was partially unclear with a deficient capsule (Fig. 3). Histopathological examination showed a tumor composed of proliferating stellate or spindle-shaped pleomorphic atypical cells on a background of myxomatous stroma and atypical cells with irregular giant nuclear or multinuclear cells. Only about 10% of viable tumor cells were observed, and most of them were found to be hemorrhagic and necrosed due to TAE. Some atypical cells were with d-Periodic acid Schiff (d-PAS-positive) cytoplasmic inclusions. Bile duct-like structures were observed in the neoplasm margin area (Fig. 4). The resection margin was too degenerated to evaluate, but atypical cells were found close to the edge. The immunohistochemical evaluation showed diffusely positive expression of vimentin; alpha 1-antichymotrypsin (α1ACT) and alpha 1-antitrypsin (α1AT); and focally positive expression of desmin, α-smooth muscle actin (α-SMA), glypican-3, and discovered on the gastrointestinal stromal tumor-1 (DOG-1) and negative expression for CAM 5.2, AE1/AE3, Hepatocyte Paraffin-1, S-100, HMB45, CD34, and c-kit (Fig. 5). Ki-67 labeling index was 40%. Based on these findings, the pathological diagnosis was UESL rather than gastrointestinal stromal tumor (GIST). The patient was discharged on postoperative day 15 as per the enhanced recovery after surgery protocols in hepatectomy [2]. Several liver metastases were observed 2 months after surgery, and sarcoma-directed chemotherapy with doxorubicin was initiated (details of chemotherapy regimens are described in Fig. 6). We changed the regimen to eribulin when there was an increase in liver metastases despite gemcitabine/docetaxel. About two years after the surgery, she felt abdominal pain again, and a CT scan was performed. One of the liver metastases grew rapidly, and an impending rupture was suspected. Palliative surgery involving partial hepatectomy and splenectomy was performed. Pathological findings showed the neoplasm with the same histopathology as the primary tumor, UESL, and Ki-67 immunohistochemistry was up to 70%. One month after the second surgery, she resumed the eribulin therapy, but the liver metastases grew gradually. The drug was changed from eribulin to trabectedin; however, 39 months after the initial surgery, one of the liver metastases increased rapidly, and a second palliative surgery with partial liver resection was performed. The ifosfamide/mesna therapy was restarted postoperatively, but the disease had progressed significantly. Forty-three months after primary operation, radiological findings revealed lung metastases. Because of neutropenia, doxorubicin and eribulin were reduced to 80% and gemcitabine/docetaxel to 75% after the second course. Trabectedin was reduced to 80% after the second course because of hepatic impairment. Pazopanib and cisplatin were used in sequence, and continued palliative care was administered; however, the patient died 49 months after the initial surgery.

## Discussion

UESL is a primary mesenchymal malignant liver neoplasm, and the concept of this neoplasm was first proposed by Stocker and Ishak in 1978 [1]. It accounts for approximately 9–13% of primary liver tumors in children [3]; however, it is extremely rare in adults, accounting for approximately 0.2% of the primary liver tumors [4]. Furthermore, UESL has a worse prognosis in adults, with a 5-year overall survival (OS) rate of 48.2% compared to 84.4% in children [5, 6]. The standard treatment strategy for UESL has not been established, and there is no drug for the treatment of UESL. However, recent studies have shown that margin-negative resection improves the OS [5, 6]. Currently, most patients receive vincristine, actinomycin-D, cyclophosphamide (VAC), or ifosfamide based on the Intergroup Rhabdomyosarcoma Study protocol [7]. To our knowledge, only 16 adult patients over the age of 18 years, including our patient, have survived for more than 48 months [3, 4, 8–19] (Table 1). With the exception of one patient who underwent liver transplantation after neoadjuvant chemotherapy [15], all the other patients received initial surgical treatment. We found that 10 patients, including our patient, who survived for more than 48 months had recurrence after the primary surgery. Of the 10 patients who relapsed, eight (80%) presented with hepatic recurrence. Although the high recurrence rate of residual liver tissue indicates that current radical resection and chemotherapy may not be sufficient to achieve complete resolution, long-term survival appeared possible even in cases with recurrence by combining surgery and chemotherapy (and/or radiation therapy). Liver transplantation for UESL has been performed to control hepatic recurrence, and there have been reports of patients who have survived for more than 10 years with the combination of chemotherapy and liver transplantation for unresectable cases [3, 20]. However, the number of such cases is small, and careful consideration and more extensive studies are required to determine the indications for combined chemotherapy and liver transplantation for adult UESL in the future.

UESL is commonly characterized by a lack of differentiation tendency, without specifically oriented differentiation, such as vessels, striated muscle, smooth muscle, fat, and nerves, and there is no specific immunohistochemical pattern [1]. In UESL, pathological findings reveal a proliferation of spindle, oval, or stellate pleomorphic immature cells with poorly defined cell borders embedded in the mucinous stroma; and near the tumor margin, entrapped bile duct-like structures surrounded by tumor cells were observed [1]. It often accompanies multinucleated giant cells and d-PAS-positive granule-containing cells [1].

In our case, immunohistochemical analysis showed diffusely positive expression of vimentin, α1ACT, and α1AT and was focally positive for desmin, α-SMA, glypican-3, and DOG-1. Local expression of myogenic markers and DOG-1 may reflect the local differentiation trend in the undifferentiated tumor. There are no prior reports on the expression of DOG-1 in UESL. DOG-1 is a member of the transmembrane protein 16 family, featured as a calcium-activated chloride channel and expressed in GIST, but the detailed functions are unknown. Recently, the details of DOG-1 have been revealed gradually by reported expression in poorly differentiated tumors [21, 22], such as sarcomatous carcinoma of the liver, in lymph node metastasis of colorectal cancer [23], and as a poor prognostic factor in breast cancer [24]. The clinical significance of DOG-1 positivity in UESL is unclear; however, it may become more evident with the accumulation of cases.

The occurrence of UESL has not been understood, but Mori et al. reported that a hamartoma-like lesion is one form of UESL and that this type of case has a good prognosis [25]. It is yet to be determined whether hamartoma becomes malignant and transforms to UESL or some UESLs present with hamartoma-like lesions. Although hamartoma-like lesions were not observed in our case, further pathological analysis and treatment development may be the key to long-term survival of patients.

## Conclusions

We verified a long-term survival case of adult UESL. Long-term survival may be achieved by combining radical surgery and multi-agent chemotherapy. Furthermore, detailed immunohistological analysis of UESL may result in more effective medications and improve OS.

**Table 1:** Previous studies’ clinical parameters of adult patients with UES who survived over 48 months

References	Author	Year	Age/sex		Follow-up (month)	Localization	Maximum diameter (cm)	Initial treatment	Resection margin	Adjuvant chemotherapy	Recurrence	Location of recurrence	Treatment for recurrence		Outcome
[8]	Grazi	1996	25/M	67.6		Right lobe	15	Surgery	Inrtaoperative tumor rupture	None	Yes	Liver		Operation	DOD
[9]	Almogy	2004	25/F	60		Right lobe	14	Surgery	Negative	Ifosfamide, doxorubicin, mesna	Yes	Liver, bone		VAC	RBS
[10]	Almogy	2005	21/F	75		Right lobe	15	Surgery	Intraoperatively, another lesion was pointed out	N.A	N.A	Intrahepatic region noted in the first surgery		Operation, ifosfamide, doxorubicin, mesna	NED
[11]	Sebastien	2005	18/F	48		Right lobe	26	Surgery	N.D	VAC	Yes	Intra-abdominal		Operation, chemotherapy	DOD
[12]	Lentz	2008	34/F	72		Right lobe	20	Surgery	Free	None	Yes	Liver, intra-abdominal		Carboplatin, etoposide, doxorubicin, ifosfamide	NED
[13]	Tsukada	2010	21/F	60		Right lobe	12	Surgery	N.D	None	No	–			NED
[14]	Kim	2011	47/F	48		Left lobe	13	Surgery	N.D	MAID	Yes	Bone		RT	NED
[15]	Dhanasekaran	2012	53/M	126		Left lobe	30	Chemotherapy → Tx	N.A	None	No	–			NED
[16]	Noguchi	2012	27/F	60		Right lobe	21	Surgery	Negative	﻿VADRCA + CDDP + RT, Peripheral stem cell extraction	No				NED
[17]	Masuda	2015	52/F	62		Right lobe	23	Surgery	N.D	None	Yes	Liver		Operation, TACE(﻿CDDP 50 mg + lipiodol 2.5 ml, epirubicin)	DOD
[17]	Masuda	2015	53/F	65		Left lobe	22	Surgery	N.D	None	Yes	Liver		Operation, TACE(﻿CDDP 80 mg + lipiodol 4 ml, 5-FU 1000 mg)	RBS
[4]	Esteban	2018	41/F	60		Right lobe	26	Surgery	Negative	None	Yes	Liver		GEM + DOC	RBS
[18]	Beksac	2018	26/F	72		Left lobe	25	Surgery	N.D	Taxol, Cisplatin, ifosfamide	No				NED
[19]	Capozza	2019	20/F	168		Right lobe	15	Surgery	Negative	VAIA	No				NED
[3]	Babu	2021	31/F	120		Left lobe	10.3	Surgery	N.D	Cyclophosphamide, vincristine	Yes	Liver		Chemotherapy (cyclophosphamide, vincristine) → Tx	NED
	Our case	2021	33/F	49		Right lobe	17	Surgery	Negative	None	Yes	Liver		Fig. 5	DOD

*UES* undifferentiated embryonal sarcoma of the liver, *Tx* transplantation, *VAC* vincristine, actinomycin-D and cyclophosphamide, *MAID* mesna, doxorubicin, ifosfamide and dacarbazine, *VAIA* vincristine, actinomycin-D, ifosfamide and doxorubicin, *VADRCA* vincristine, doxorubicin, actinomycin-D and cyclophosphamide, *CDDP* cisplatin, *RT* radiation therapy, *TACE* transcatheter arterial chemoembolization, *5-FU* 5-fluorouracil, *GEM* gemcitabine, *DOC* docetaxel, *ND* not documented, *NA* not applicable, *NED* no evidence of disease, *DOD* died of disease, *RBS* recurrence but survived

## Data Availability

All data generated or analyzed during this study are included in this published article. Further material and information on this case report are available from the corresponding author on reasonable request.

## Abbreviations

UESL: Undifferentiated embryonal sarcoma of the liver
CT: Computed tomography
TAE: Transcatheter arterial embolization
d-PAS: D-Periodic acid Schiff
GIST: Gastrointestinal stromal tumor
DOG-1: Discovered on gastrointestinal stromal tumor -1
OS: Overall survival
α1ACT: Alpha 1-antichymotrypsin
α1AT: Alpha 1-antitrypsin
α-SMA: α-Smooth muscle actin
